# Reaction times match IQ for major causes of mortality: Evidence from a population based prospective cohort study

**DOI:** 10.1016/j.intell.2018.05.005

**Published:** 2018

**Authors:** Geoff Der, Ian J. Deary

**Affiliations:** a MRC/CSO Social and Public Health Sciences Unit, University of Glasgow, UK; b Centre for Cognitive Ageing and Cognitive Epidemiology, University of Edinburgh, UK

## Abstract

**Introduction:**

The association of premorbid cognitive ability with all-cause mortality is now well established. However, since all-cause mortality is relatively uninformative about aetiology, evidence has been sought, and is beginning to accumulate, for associations with specific causes of mortality. Likewise, the underlying causal pathways may be illuminated by considering associations with different measures of cognitive ability. For example, critics of IQ type measures point to possible cultural or social biases and there is, consequently, a need for more culturally neutral measures such as reaction times. We examine the associations of cognitive ability with major causes of mortality, including: cardiovascular disease, cancer and respiratory disease and compare the results for a standard IQ test, the Alice Heim 4 (AH4), with those for simple and four-choice reaction times.

**Methods:**

Data were derived from the oldest cohort of the West of Scotland Twenty-07 Study. Participants were randomly sampled from the Central Clydeside Conurbation, a mainly urban area centred on Glasgow city. At baseline, aged 56, they were interviewed in their homes by trained interviewers; the AH4 was administered and reaction times measured using a portable electronic device. Vital status was ascertained via linkage to the NHS central register. Cox regression was used in SAS 9.4 for the main analyses. Adjustments were made for sex, smoking status and social class.

**Results:**

Full data on AH4, RT and covariates were available for 1350 out of 1551. During 29 years of follow-up, there were 833 deaths: 279 cardiovascular disease (CVD) (168 CHD; 68 stroke); 291 cancer; 97 respiratory disease; 42 digestive disease; and 39 dementia. The 85 remaining deaths were a heterogeneous mixture with no cause accounting for more than 14.

AH4 scores were associated with most major causes. Digestive disease and dementia had similar effect sizes but were not significant. Within cardiovascular disease, there was an association with coronary heart disease but not stroke. The association with cancer was primarily due to those cancers related to smoking.

RT measures were mostly associated with the same causes of death. Where significant, effects were in the same directions and of similar magnitude. That is, lower AH4 scores, longer reaction times, and more variable reaction times were all associated with increased mortality risk from the major causes of death. A summary measure of RT outperformed the AH4 for most causes.

**Conclusion:**

The association between intelligence with mortality from the major causes is also seen with reaction times. That effect sizes are of similar magnitude is suggestive of a common cause. It also implies that the association of cognitive ability with mortality is unlikely to be due to any social, cultural or educational biases that are sometimes ascribed to intelligence measures.

## Highlights

• Intelligence and reaction times are both associated with most major causes of mortality
• Effect sizes are of similar magnitude
• A summary RT measure provides the more parsimonious explanation for most causes

## 1 Introduction

The association of premorbid intelligence with all-cause mortality is increasingly well established. A meta-analytic summary of 16 studies (Calvin et al., 2010) showed a 24% (95% CI: 23%,25%) lower mortality risk per standard deviation of intelligence score. However, all-cause mortality is relatively uninformative about aetiology and more evidence is needed for specific causes, particularly the major causes of death. Until recently evidence for associations with specific causes was limited to a few causes. Among the major causes a number of studies supported an association with cardiovascular disease (Batty, Shipley, Gale, Mortensen, & Deary, 2008; Batty et al., 2009b; Christensen, Mortensen, Christensen, & Osler, 2016; Carole L. Hart et al., 2003; Carole L Hart et al., 2004; Hemmingsson, v Essen, Melin, Allebeck, & Lundberg, 2007). Among minor causes there was evidence for associations with some external causes of death, such as suicide and injuries (Batty, Gale, Tynelius, Deary, & Rasmussen, 2009a; Christensen et al., 2016; Osler, Andersen, Laursen, & Lawlor, 2007). Evidence for dementia was suggestive although numbers were small (McGurn, Deary, & Starr, 2008; Whalley et al., 2000).

More recently these findings have been reinforced and extended by the results of a study that followed up a national year of birth cohort from age 11 to 79 (Calvin et al., 2017). In 25,143 deaths out of 65,765 individuals there were associations between childhood intelligence measured at age 11 and all major causes of death, including cardiovascular disease (CVD), cancer, respiratory disease, digestive disease and dementia. Among CVD deaths the overall association was mirrored for deaths from coronary heart disease and stroke. However, the association found for cancer deaths was confined to those cancers related to smoking. The study also found associations with deaths from external causes and from injury but not from intentional self-harm, although self-harm did have by far the smallest number of deaths. For the significant associations hazard ratios per standard deviation higher intelligence score were in the range .72 to .84, corresponding to a reduction in mortality risk of between 28% and 16%.

The same study (Calvin et al., 2017) also reported results from a replication sample, the oldest cohort of the West of Scotland Twenty-07 study, where intelligence was assessed in middle age. The results were very similar with the exception of stroke which showed little evidence of an association in the Twenty-07 study. Here, in a further report on the Twenty-07 study, we present essentially the same data on intelligence test scores in order to compare the results with those for reaction time parameters.

There are a number of reasons why such a comparison is of interest. One hypothesis that has been proposed to explain why intelligence might be related to mortality is that intelligence tests assess some aspect of bodily integrity that affects the efficiency of information processing (Deary, 2012; Whalley & Deary, 2001). Reaction times can be considered as simpler and more fundamental measures of information processing than intelligence tests are. They make little, if any, reliance on prior knowledge and so are less likely to be influenced by educational or social background. Equally, any cultural specificity is minimal. Reaction times are moderately correlated with intelligence both in this sample (Deary, Der, & Ford, 2001; Der & Deary, 2017) and more generally (Sheppard & Vernon, 2008). In a previous analysis on the Twenty-07 study that was restricted to all cause mortality (when there were too few deaths to examine specific causes) we found that scores on part I of the Alice Heim 4 (AH4) and reaction time measures were both strongly related to mortality and that reaction time data could provide a more parsimonious explanation (Deary and Der, 2005a, Deary and Der, 2005b).

We are only aware of two previous studies that have examined the association between reaction times and the major causes of mortality (Hagger-Johnson, Deary, Davies, Weiss, & Batty, 2014; Shipley, Der, Taylor, & Deary, 2008). The study by Hagger-Johnson et al had limited coverage of reaction times and of causes of mortality. Only simple reaction time was measured and causes of mortality were limited to cardiovascular disease and cancer. The study by Shipley et al. was more extensive: both simple and 4-choice reaction times were measured and a wider range of causes of mortality covered. They also included measures of memory and visuospatial reasoning. However, these two measures were simple scores out of 10 or 6, respectively, and so did not permit a direct comparison of effect sizes for reaction times with general mental ability.

Our aim here is to examine the associations of simple and four choice reaction time mean and variability with mortality from the major causes and to compare the results with those for a standard measure of general mental ability.

## 2 Methods

### 2.1 Participants

Participants are drawn from the West of Scotland Twenty-07 Study. This is a longitudinal study designed to investigate the social determinants of health and health inequalities. It comprises three narrow age cohorts who were aged 15, 35 and 56 at baseline in 1987/88. Study participants were randomly sampled from the Central Clydeside Conurbation, a mainly urban area centred on Glasgow city. They were interviewed in their homes by trained interviewers at baseline and on up to four further occasions over the following 20 years. Further details of the study are given elsewhere (Benzeval et al., 2008). A comparison with data from the 1991 UK Census showed it to be representative of the underlying population in terms of sex, social class, car ownership and household tenure (Der, 1998).

Data used here pertain to the oldest of the three cohorts obtained at baseline plus vital status obtained by record linkage.

### 2.2 Measures

#### 2.2.1 Alice Heim 4 test of General Intelligence (AH4)

Part I of the AH4 (Heim, 1970) was administered to the oldest cohort of the Twenty-07 study at baseline. The AH4 was designed for use with a cross section of the adult population and part I consists of 65 questions which measure verbal and numeric cognitive abilities. It was administered according to the instructions in the test manual. The result used here is the total number of questions answered correctly within the time limit of 10 minutes. For comparability with the reaction time measures this total score is standardised to zero mean and unit standard deviation (z-scored) and the sign of this reversed.

### 2.3 Reaction time measures

Simple and four choice reaction times were measured using a portable electronic device. Full details, including a diagram of the device are given elsewhere (Deary et al., 2001). The simple RT task involves 8 practise trials followed by 20 test trials. The four choice task has 8 practise trials and 40 test trials. The device does not store the results of individual trials but calculates the mean and standard deviation of the test trials. For the four choice task, means and standard deviations are recorded separately for correct and incorrect responses along with the number of errors. Measures used here are the mean and standard deviation of the simple reaction times and of the correct responses to the choice RT task. One person with excessive errors (12) was excluded. The four resulting RT measures were standardised to z-scores for comparability with the AH4.

### 2.4 Vital status

Vital status was ascertained via linkage to the UK's NHS Central Register. Participants of the Twenty-07 study were ‘flagged’ at the NHS Central Register at the outset of the study and since then the register has provided regular notifications of deaths. These notifications include date and cause of death. Primary and secondary causes are given in textual and coded forms, the latter being a mixture of ICD versions 9 and 10. The primary outcome here is the major cause of death. The latest recorded death in the data set was on the 16th August 2017 and all those alive were censored on that date. The NHS central register also provides information on embarkations (emigration) and re-entries. Those known to have emigrated were censored at the date of embarkation unless a later re-entry date or death notification was given.

Causes of death are categorised in terms of ICD codes using the same categories as for the study by Calvin et al. (2017). The ICD 9 and ICD-10 codes used to form these categories are given in Table A of the Appendix A to that paper.

### 2.5 Covariates

Smoking status was classified as current smoker/ex-smoker/never smoked. The social class measure is derived from the occupation of the head of household coded to the six fold Registrar General's classification (OPCS, 1980) and dichotomised into manual vs non-manual.

### 2.6 Statistical analysis

Cox proportional hazards regression was used for the main analyses. The hazard to be modelled is the mortality risk from all causes or from specific causes. In a Cox regression the log of the hazard is expressed as an additive function of the predictor variables. The regression coefficients, when antilogged, give hazard ratios and these are estimates of the proportionate change in mortality risk per unit change in the predictor. Hazard ratios above 1 indicate that the mortality risk is higher for greater values of the predictor and hazard ratios below 1 indicate lower mortality risk. More specifically, subtracting 1 from the hazard ratio and multiplying the result by 100 [(HR-1) × 100] gives the percentage change in the mortality risk for a 1 unit increase in the predictor. A hazard ratio of 1 represents a null effect where the mortality risk is unrelated to the predictor. Hence, attenuation of a hazard ratio, for example after adjusting for covariates, is represented by a change in the hazard ratio bringing it closer to the value of 1.

To make the results directly comparable between the cognitive variables we standardized the AH4 scores and reaction time measures to a mean of zero and unit standard deviation, ie to z-scores, and reversed the sign of the standardized AH4 scores. Thus, a hazard ratio above one represents the proportionate increase in mortality risk per standard deviation *lower* performance on the cognitive tasks.

We fit a series of models: first each of the cognitive variables is modelled individually both unadjusted and then adjusted for covariates (sex, smoking and SES); then the cognitive variables are modelled jointly in two further models, both adjusted for covariates. In the first of these joint models all five cognitive variables are entered in the model simultaneously; in the second of the joint models backwards elimination is applied to the cognitive variables to retain a parsimonious model. The covariates are retained in this latter, ‘selected’, model. This process is repeated for all-cause mortality and each of the specific causes.

In a further set of analyses, we replaced the four individual RT measures with a single summary measure of RT, the first principal component score.

Additional analyses, presented in an Appendix A, were made for cancers split into lung cancer and all other cancers to allow more direct comparison with the results of Shipley et al. (2008) and with adjustment for individual covariates.

All analyses were carried out using SAS version 9.4.

### 2.7 Results

Full data on AH4, RT and covariates were available for 1350 out of 1551 (87%). These comprised 741 women and 609 men with a mean age of 56.2 years (SD 0.63) at baseline. During 29 years of follow-up, there were 833 deaths: 279 from cardiovascular disease (coronary heart disease 168, stroke 68), 291 from cancers (189 smoking related), 97 from respiratory disease, 42 digestive diseases, 39 Dementia and 85 from all other causes. The other causes were heterogeneous with no group large enough to analyse; the largest group was infectious diseases with N=14. There were only 10 deaths from external causes.

Table 1 presents descriptive statistics for the cognitive measures and covariates broken down by vital status. Those who died during follow up were more likely to be male, manual social class, and current smokers. They also had lower AH4 scores and longer and more variable reaction times. All of these differences were significant at p<.0001 with the exception of Choice RT SD (P<.03). The standardized mean differences were .33 for AH4 and CRT mean. For SRT mean, SRT SD and CRT SD the standardized mean differences were and .27, .23 and .12, respectively.

**Table 1:** Descriptive statistics by vital status.

		Alive	Died	Total
	N	517	833	1350
	%	38	62	100
Sex				
Female	N	334	407	741
	%	45	55	100
Male	N	183	426	609
	%	30	70	100
Social Class				
Non-manual	N	264	310	574
	%	46	54	100
Manual	N	253	523	776
	%	33	67	100
Smoking Status				
Never Smoked	N	255	195	450
	%	57	43	100
Ex-smoker	N	116	191	307
	%	38	62	100
Current smoker	N	146	447	593
	%	25	75	100
AH4	Mean	29	25	27
	SD	11	11	12
CRT mean	Mean	703	744	728
	SD	103	118	114
SRT mean	Mean	338	374	360
	SD	103	135	125
CRT SD	Mean	129	134	132
	SD	34	38	37
SRT SD	Mean	84	100	94
	SD	59	69	66

The correlation matrix for covariates and cognitive measures is given in Appendix A Table A1. Correlations between the (reversed) AH4 scores and the four RT measures were: CRT mean, 0.50; CRT SD; 0.28; SRT mean, 0.33; SRT SD, 0.29. For the RT summary score the correlation was .46.

Table 2 shows the results of the models assessing the association between the cognitive measures and mortality. Models are included for all-cause mortality and the major causes of death, namely: cardiovascular disease (CVD), cancer, respiratory disease, digestive disease and dementia. For CVD results are also given for two important subgroups: coronary heart disease (CHD) and stroke. Cancers deaths are additionally subdivided into those related to smoking versus all other cancers.

**Table 2:** Hazard ratios, 95% CIs, and p values for risk of mortality per standard deviation disadvantage in AH4 score and reaction times.

		Unadjusted			Sex, smoking, SES adjusted			Fully adjusted			Selected		
Cause of Death	Predictor	Hazard ratio	95% CI	p	Hazard ratio	95% CI	p	hazard ratio	95% CI	p	Hazard ratio	95% CI	p
All Cause	AH4	1.29	1.20 to 1.38	<.001	1.23	1.13 to 1.33	<.001	1.13	1.03 to 1.23	0.008	1.13	1.03 to 1.24	0.008
	CRT mean	1.30	1.22 to 1.38	<.001	1.24	1.15 to 1.33	<.001	1.08	0.96 to 1.21	0.186	1.13	1.04 to 1.23	0.005
	SRT mean	1.23	1.15 to 1.31	<.001	1.21	1.14 to 1.30	<.001	1.08	0.97 to 1.20	0.151			
	CRT SD	1.12	1.05 to 1.20	0.001	1.11	1.03 to 1.19	0.003	1.02	0.94 to 1.11	0.632			
	SRT SD	1.18	1.11 to 1.26	<.001	1.18	1.10 to 1.26	<.001	1.06	0.97 to 1.16	0.178	1.10	1.02 to 1.18	0.015
CVD	AH4	1.30	1.15 to 1.48	<.001	1.27	1.10 to 1.47	<.001	1.19	1.02 to 1.39	0.027	1.22	1.05 to 1.41	0.008
	CRT mean	1.29	1.15 to 1.44	<.001	1.22	1.08 to 1.39	0.002	1.01	0.83 to 1.23	0.947			
	SRT mean	1.21	1.08 to 1.35	<.001	1.20	1.07 to 1.35	0.002	1.09	0.91 to 1.31	0.324	1.14	1.01 to 1.29	0.037
	CRT SD	1.13	1.01 to 1.27	0.039	1.16	1.03 to 1.30	0.013	1.09	0.95 to 1.25	0.221			
	SRT SD	1.16	1.03 to 1.30	0.011	1.16	1.04 to 1.31	0.011	1.04	0.89 to 1.21	0.640			
CHD	AH4	1.35	1.15 to 1.59	<.001	1.30	1.08 to 1.56	0.006	1.17	0.96 to 1.43	0.119	1.24	1.03 to 1.49	0.023
	CRT mean	1.35	1.18 to 1.55	<.001	1.29	1.11 to 1.51	<.001	1.05	0.82 to 1.34	0.713			
	SRT mean	1.25	1.09 to 1.43	0.002	1.23	1.07 to 1.42	0.005	1.07	0.86 to 1.35	0.539			
	CRT SD	1.19	1.03 to 1.38	0.020	1.22	1.06 to 1.41	0.006	1.12	0.94 to 1.34	0.189	1.18	1.02 to 1.36	0.030
	SRT SD	1.21	1.05 to 1.39	0.008	1.21	1.05 to 1.40	0.008	1.07	0.88 to 1.30	0.502			
Stroke	AH4	1.08	0.84 to 1.38	0.551	1.01	0.76 to 1.33	0.968	1.00	0.73 to 1.35	0.975			
	CRT mean	1.10	0.85 to 1.43	0.469	1.02	0.76 to 1.37	0.897	1.00	0.66 to 1.53	0.984			
	SRT mean	1.08	0.83 to 1.40	0.589	1.06	0.81 to 1.39	0.672	1.07	0.72 to 1.59	0.724			
	CRT SD	0.95	0.73 to 1.24	0.716	0.97	0.75 to 1.26	0.821	0.96	0.71 to 1.30	0.802			
	SRT SD	1.02	0.79 to 1.32	0.875	1.02	0.78 to 1.33	0.871	0.99	0.71 to 1.38	0.948			
Cancer: all	AH4	1.20	1.07 to 1.36	0.002	1.13	0.99 to 1.30	0.073	1.12	0.96 to 1.30	0.142			
	CRT mean	1.13	0.99 to 1.27	0.061	1.05	0.92 to 1.21	0.449	0.91	0.75 to 1.11	0.351			
	SRT mean	1.18	1.05 to 1.32	0.005	1.16	1.03 to 1.30	0.015	1.16	0.97 to 1.38	0.106	1.16	1.03 to 1.30	0.015
	CRT SD	1.02	0.90 to 1.15	0.751	0.99	0.87 to 1.12	0.828	0.98	0.85 to 1.12	0.733			
	SRT SD	1.13	1.01 to 1.27	0.034	1.12	1.00 to 1.26	0.059	1.04	0.90 to 1.21	0.584			
Cancer: smoking	AH4	1.28	1.10 to 1.49	0.001	1.13	0.96 to 1.35	0.150	1.11	0.92 to 1.34	0.290			
	CRT mean	1.18	1.02 to 1.37	0.025	1.06	0.90 to 1.26	0.466	0.88	0.69 to 1.12	0.310			
	SRT mean	1.20	1.04 to 1.37	0.010	1.17	1.01 to 1.35	0.036	1.12	0.91 to 1.39	0.293			
	CRT SD	1.09	0.94 to 1.26	0.270	1.04	0.90 to 1.20	0.616	1.03	0.87 to 1.22	0.726			
	SRT SD	1.20	1.05 to 1.37	0.008	1.18	1.03 to 1.36	0.018	1.13	0.94 to 1.35	0.200	1.18	1.03 to 1.36	0.018
Cancer: other	AH4	1.08	0.88 to 1.32	0.453	1.13	0.90 to 1.41	0.291	1.13	0.88 to 1.46	0.322			
	CRT mean	1.02	0.81 to 1.27	0.891	1.04	0.83 to 1.31	0.732	0.97	0.70 to 1.35	0.875			
	SRT mean	1.13	0.93 to 1.38	0.229	1.14	0.93 to 1.40	0.199	1.23	0.91 to 1.66	0.182			
	CRT SD	0.90	0.72 to 1.12	0.331	0.88	0.70 to 1.11	0.275	0.86	0.67 to 1.12	0.266			
	SRT SD	0.99	0.80 to 1.23	0.962	1.00	0.80 to 1.24	0.972	0.89	0.67 to 1.18	0.415			
Respiratory	AH4	1.52	1.23 to 1.88	<.001	1.38	1.08 to 1.77	0.010	1.16	0.88 to 1.52	0.298			
	CRT mean	1.49	1.27 to 1.75	<.001	1.43	1.18 to 1.72	<.001	1.41	1.03 to 1.94	0.034	1.27	1.03 to 1.57	0.028
	SRT mean	1.33	1.12 to 1.58	0.001	1.30	1.09 to 1.56	0.004	0.89	0.67 to 1.20	0.447			
	CRT SD	1.10	0.90 to 1.35	0.336	1.05	0.86 to 1.29	0.628	0.84	0.66 to 1.06	0.148			
	SRT SD	1.38	1.18 to 1.63	<.001	1.38	1.17 to 1.64	<.001	1.31	1.05 to 1.64	0.019	1.26	1.04 to 1.54	0.020
Digestive	AH4	1.33	0.97 to 1.83	0.079	1.33	0.92 to 1.91	0.127	1.21	0.81 to 1.79	0.354			
	CRT mean	1.32	0.99 to 1.75	0.055	1.28	0.95 to 1.74	0.109	0.92	0.57 to 1.50	0.746			
	SRT mean	1.20	0.90 to 1.61	0.212	1.19	0.88 to 1.62	0.247	0.95	0.59 to 1.51	0.814			
	CRT SD	1.41	1.09 to 1.83	0.010	1.40	1.08 to 1.82	0.012	1.35	0.97 to 1.87	0.073	1.40	1.08 to 1.82	0.012
	SRT SD	1.33	1.03 to 1.71	0.031	1.33	1.02 to 1.73	0.032	1.28	0.91 to 1.80	0.158			
Dementia	AH4	1.24	0.89 to 1.72	0.198	1.06	0.73 to 1.53	0.750	0.98	0.66 to 1.47	0.940			
	CRT mean	1.28	0.94 to 1.74	0.119	1.20	0.85 to 1.69	0.304	1.27	0.74 to 2.19	0.379			
	SRT mean	1.17	0.83 to 1.63	0.369	1.12	0.79 to 1.59	0.512	1.05	0.62 to 1.77	0.854			
	CRT SD	1.07	0.77 to 1.49	0.675	1.01	0.72 to 1.42	0.963	0.91	0.61 to 1.36	0.662			
	SRT SD	1.02	0.72 to 1.45	0.900	1.00	0.70 to 1.42	0.991	0.90	0.57 to 1.43	0.663			

Notes: Unadjusted and covariate adjusted results are from separate models for each cognitive variable. The fully adjusted results are for a single model (per cause) that includes the covariates and all cognitive variables. The Selected model results are derived from the fully adjusted model by backwards elimination of non-significant cognitive variables whilst retaining the covariates.

AH4 scores are reversed.

In unadjusted analyses, AH4 scores were associated with all-cause mortality and most of the major causes, namely: CVD, cancer, and respiratory disease. Digestive disease and dementia had similar effect sizes but were not significant. Among CVD deaths there were associations with CHD but not with stroke. Among cancer deaths the association was significant for those related to smoking but not for the remaining cancers. Hazard ratios were in the range 1.28 (smoking related cancers) to 1.52 (respiratory diseases), representing between 28% and 52% increase in mortality risk for one standard deviation (12 points) lower score.

Reaction time measures showed broadly similar patterns of association to that of the AH4 scores. Most measures were significantly associated with the same causes of death: CVD, CHD, smoking-related cancers, and respiratory disease. Effect sizes for CRT mean were very similar to those for AH4. With the exception of cancer mortality HRs were within +/- .04 of those for AH4. Effects sizes for SRT mean were generally somewhat weaker, albeit significant for the same causes. Effects sizes for SRT SD tended to be weaker again but also significant for the same causes. CRT SD had the weakest associations with mortality and those for smoking related cancer and respiratory disease were non-significant but it was significantly related to mortality from digestive disease with an HR of 1.41 (1.09, 1.83).

After adjustment for covariates effect sizes were attenuated to varying degrees but most of those that were significant in unadjusted analyses remained so after adjustment for covariates. Exceptions were the associations of AH4 and CRT mean with smoking related cancers which were both reduced to non-significance after adjustment for covariates. For cancers overall the same was true of AH4 and SRT SD. Appendix A Table A2 shows the impact of adjusting for covariates individually. The main point of note there is that the effect sizes for CRT SD are generally increased after adjustment for sex. That is, sex acts as a negative confounder, or suppressor, of the association between CRT SD and mortality because women have more variable CRT but tend to live longer.

The fully adjusted models include all cognitive variables simultaneously plus the covariates. Hence, the results given are from a single model per cause in contrast to the unadjusted and covariate adjusted results where the results are from separate models, i.e. one for each cognitive measure. The focus is on the extent of attenuation when cognitive variables are mutually adjusted. For all causes, CVD and CHD all effects are considerably attenuated, mostly by around a half to two thirds. While AH4 remains significant for all-cause and CVD, all other effects are non-significant. For cancer overall, smoking related cancers and digestive diseases all effects that remained significant after adjustment for covariates were attenuated to non-significance. For respiratory disease, AH4 and SRT mean were attenuated to non-significance whereas CRT mean and SRT SD were attenuated to much lesser degrees and remained significant.

The final set of (‘selected’) results are derived from the ‘fully adjusted’ model by backwards elimination from among the cognitive variables. The models are constrained so that the covariates are retained in all cases. For all causes, CVD and CHD, the AH4 score is retained together with one or two of the RT measures. For cancer overall, smoking related cancer, respiratory and digestive diseases one or two of the RT measures are retained but not AH4. The RT measure, or measures, that are retained differs between causes.

For the purposes of a more direct comparison with the results of Shipley et al. (2008) results for cancer broken down into lung cancer and all other cancers are given in Appendix A Table A3.

Table 3 shows the results obtained when the RT summary measure is used in place of the four separate RT measures. For AH4 the results for the unadjusted and covariate adjusted models are duplicated from Table 2 for ease of comparison. In unadjusted results HRs for the RT summary are of similar magnitude to AH4 scores albeit mostly slightly smaller. For covariate adjusted models the results are much the same as for the individual RT measures. That is, there is some attenuation but significant effects remain so. With a single measures of RT, the results for RT and AH4 in the fully adjusted model are more comparable. Both remain significant for all-cause and CVD; only RT remains significant for CHD and respiratory disease; and neither is significant for cancer related to smoking and for digestive disease. Where one or both are significant the results carry forward into the selected model. For the last two causes where neither was significant when mutually adjusted, the RT summary is retained in the selected model.

**Table 3:** Hazard ratios, 95% CIs, and p values for risk of mortality per standard deviation disadvantage in AH4 score and reaction time summary score

		Unadjusted			Sex, smoking, SES adjusted			Fully adjusted			Selected		
Cause of death	Predictor	Hazard ratio	95% CI	p	Hazard ratio	95% CI	p	Hazard ratio	95% CI	p	Hazard ratio	95% CI	p
All Cause	AH4	1.29	1.20 to 1.38	<.001	1.23	1.13 to 1.33	<.001	1.13	1.04 to 1.23	0.006	1.13	1.04 to 1.23	0.006
	RT PC1	1.27	1.20 to 1.35	<.001	1.24	1.16 to 1.32	<.001	1.19	1.11 to 1.28	<.001	1.19	1.11 to 1.28	<.001
CVD	AH4	1.30	1.15 to 1.48	<.001	1.27	1.10 to 1.47	<.001	1.18	1.02 to 1.38	0.031	1.18	1.02 to 1.38	0.031
	RT PC1	1.25	1.13 to 1.39	<.001	1.23	1.11 to 1.38	<.001	1.17	1.04 to 1.32	0.011	1.17	1.04 to 1.32	0.011
CHD	AH4	1.35	1.15 to 1.59	<.001	1.30	1.08 to 1.56	0.006	1.17	0.96 to 1.42	0.125			
	RT PC1	1.32	1.16 to 1.49	<.001	1.30	1.14 to 1.48	<.001	1.23	1.06 to 1.43	0.005	1.30	1.14 to 1.48	<.001
Stroke	AH4	1.08	0.84 to 1.38	0.551	1.01	0.76 to 1.33	0.968	1.00	0.74 to 1.34	0.978			
	RT PC1	1.06	0.82 to 1.36	0.678	1.03	0.78 to 1.34	0.847	1.03	0.77 to 1.37	0.849			
Cancer: all	AH4	1.20	1.07 to 1.36	0.002	1.13	0.99 to 1.30	0.073	1.10	0.95 to 1.27	0.214			
	RT PC1	1.15	1.03 to 1.29	0.013	1.11	0.99 to 1.25	0.080	1.08	0.95 to 1.23	0.265			
Cancer: smoking	AH4	1.28	1.10 to 1.49	0.001	1.13	0.96 to 1.35	0.150	1.08	0.89 to 1.30	0.434			
	RT PC1	1.22	1.07 to 1.39	0.003	1.15	1.00 to 1.33	0.048	1.13	0.96 to 1.31	0.134	1.15	1.00 to 1.33	0.048
Cancer: other	AH4	1.08	0.88 to 1.32	0.453	1.13	0.90 to 1.41	0.291	1.14	0.89 to 1.45	0.305			
	RT PC1	1.02	0.83 to 1.26	0.842	1.03	0.83 to 1.28	0.776	0.98	0.78 to 1.25	0.898			
Respiratory	AH4	1.52	1.23 to 1.88	<.001	1.38	1.08 to 1.77	0.010	1.20	0.92 to 1.57	0.173			
	RT PC1	1.43	1.22 to 1.67	<.001	1.38	1.17 to 1.63	<.001	1.30	1.08 to 1.57	0.005	1.38	1.17 to 1.63	<.001
Digestive	AH4	1.33	0.97 to 1.83	0.079	1.33	0.92 to 1.91	0.127	1.16	0.78 to 1.71	0.465			
	RT PC1	1.38	1.08 to 1.76	0.009	1.37	1.06 to 1.76	0.015	1.31	0.98 to 1.73	0.065	1.37	1.06 to 1.76	0.015
Dementia	AH4	1.24	0.89 to 1.72	0.198	1.06	0.73 to 1.53	0.750	1.02	0.69 to 1.51	0.908			
	RT PC1	1.18	0.87 to 1.62	0.292	1.11	0.79 to 1.56	0.539	1.10	0.77 to 1.59	0.596			

Notes: Unadjusted and covariate adjusted results are from separate models for AH4 and RT. The fully adjusted results are for a single model (per cause) that includes the covariates, AH4 and the RT summary score. The Selected model results are derived from the fully adjusted model by backwards elimination whilst retaining the covariates.

AH4 unadjusted and covariate adjusted results are duplicated from Table 2 to facilitate comparison.

AH4 scores are reversed.

## 3 Discussion

In this sample of community dwelling adults drawn from a predominantly urban area in the West of Scotland, and born in the early 1930's, cognitive ability assessed at age 56 was associated with mortality from the major causes over the following 29 years. With the exception of stroke, effect sizes for the AH4 score were similar to those obtained for a whole population sample born in Scotland in 1936 and assessed in childhood using the Moray House test No. 12. The study reported here also included simple and 4 choice reaction time measures. These were mostly associated with the same causes of death as the AH4 scores and in many cases effect sizes were of similar magnitude. Effects were only partially attenuated when adjusted for important covariates. When mutually adjusted, in addition, attenuation was much greater. Selecting a more parsimonious subset from the cognitive measures yielded results that differed between causes. When the four RT measures were combined as a principal component score this tended to be chosen in preference to the AH4 score in selected models.

A number of previous studies have reported associations between reaction time measures and all-cause mortality finding elevated mortality risk associated with longer and more variable reaction times (Batterham, Bunce, Mackinnon, & Christensen, 2014; Deary and Der, 2005a, Deary and Der, 2005b; Hagger-Johnson et al., 2014; Metter, Schrager, Ferrucci, & Talbot, 2005; Shipley, Der, Taylor, & Deary, 2006; Yamada, Shimizu, Kasagi, & Sasaki, 2013).

To our knowledge, only two previous studies have reported reaction time's associations with cause specific mortality for the major causes of death. These are the NHANES-III study (Hagger-Johnson et al., 2014) and the UK Health and Lifestyle Survey (HALS) (Shipley et al., 2008).

The NHANES-III study included a measure of simple reaction time and examined associations of SRT mean and variability with mortality for 104 CVD deaths and 84 cancer deaths. They found significant associations for CVD but not cancer; the hazard ratios per standard deviation for CVD were: SRT mean 1.36 (1.17,1.58) and SRT SD 1.50 (1.33,1.70). For cancer they were: SRT mean 0.85 (0.54,1.34) and SRT SD 0.99 (0.72,1.34). The study did not include a measure of CRT.

The UK HALS study is more directly comparable to the present study since the two studies used the same RT equipment and protocol. During 21 years of follow-up of the HALS 1,550 deaths occurred and for analysis these were grouped into CVD (including CHD and stroke), respiratory disease, lung cancer and all other cancers. In their baseline model (adjusted for age and sex), there were significant associations with all RT measures for CVD, CHD, and respiratory disease. All RT measures, except CRT SD, were significantly associated with Stroke. For cancer there was only one significant association: that between CRT mean and lung cancer. For all causes, the effect size was greatest for CRT mean with HRs from 1.19 (CVD, CHD), through 1.20 (Lung cancer), 1.21 (respiratory) to 1.29 (stroke).

To sum up, there is agreement between the three studies for an association between longer and more variable reaction times and greater risk of death from CVD, albeit that NHANES-III only has data on SRT. For cancer, the only other cause common to all three studies, there is little evidence for an association with cancers taken as a whole but stronger evidence for smoking related cancers, such as lung cancer.

There are differences between the current study and the HALS and NHANES-III studies which may be relevant to the differences in findings. The most notable difference is the age ranges of the samples. The present study uses a narrow age cohort whereas HALS covers the whole adult age range (18-97 years) and NHANES-III covers the ages 20-59. However, the impact of age is not clear. For all-cause mortality the association with RT in the HALS study was strongest in the youngest age group (Shipley et al., 2006), whereas there was no clear pattern in NHANES-III(Hagger-Johnson et al., 2014). The latter also produced estimates for CVD and cancer by three age groups but these were inevitably based on small numbers.

As well as differences in the age ranges covered there are also differences in mean age and length of follow up. NHANES-III has a mean age of 37 with 15 years follow up; HALS mean age of 45 with 21 years of follow up and this study a mean age of 56 with 29 years follow up.

Neither of the previous two studies afforded a direct comparison of RT effects with a measure of general intelligence. NHANES-III only reported SRT. Although the HALS study did report associations with measures of verbal memory and visuospatial reasoning, as these two measures were relatively crude - a score out of 10 or 6, respectively – the effect sizes were not directly comparable with those for the RT measures. In contrast, this study allows direct comparison and is, to our knowledge, the first to do so for the major causes of death.

Comparison of the effect sizes between the RT measures and AH4 scores has potential implications for their interpretation. We have already noted elsewhere the marked similarity between the results for AH4 scores in this study and those for a whole nation sample (Calvin et al., 2017). We have also noted the similarity in effect sizes for RT measures and AH4 scores in their relationship to all-cause mortality (Deary and Der, 2005a, Deary and Der, 2005b). From the results here, it is clear that this similarity in effect sizes extends across the major causes. An important point to note is that the effect sizes are more similar than would be expected simply in virtue of the correlation between the RT measures and AH4 scores, the largest of which is 0.5 for CRT mean. A parsimonious explanation for this would be that it is some common component which is particularly associated with mortality. This component could be a, largely genetic, ‘fitness factor’ {Arden, 2009; Arden, 2016} or it could simply reflect the g-loading of the measures. Alternatively, the results are also compatible with theories that stress the fundamental role of information processing speed, indexed by RT, both in general mental ability (Jensen, 1982) and in cognitive ageing (Salthouse, 1996).

The variation in effect sizes across different causes is more difficult to explain. In the HALS study CRT mean consistently had the largest effect size but, although it does tend to be among the larger effects here, there is not the same consistency. Clearly, some causes of death are more preventable than others and hence more likely to show an association; the contrast between cancers related or unrelated to smoking is the obvious example. However, it is not clear why this would affect the cognitive measures differentially. A possibility that cannot be ruled out is that the variation is simply due to random effects of measurement error. In a Cox model biased effect estimates can result from misclassification of the cause of death as well as from measurement error in the predictors{Van Rompaye, 2012}.

A contrary view posits that variability in reaction times, whether trial to trial variability within a task (as in RT SD) or task to task variability (such as test-retest reliability), do not only reflect measurement error but are indicators of systematic underlying, usually pathological, processes (Bunce, MacDonald, & Hultsch, 2004). The proponents of this theory point to increased RT variability associated with a number of neurological conditions (Hetherington, Stuss, & Finlayson, 1996) and to studies where RT SD is more predictive than mean RT (Haynes, Bauermeister, & Bunce, 2017). The results here are not consistent enough across causes to provide evidence either for or against this hypothesis.

The clear anomaly among our results is the absence of an association between AH4 or any of the RT measures and stroke mortality. This is in contrast to the findings of Calvin et al. (2010) where the association of intelligence with stroke mortality was very similar to that for CHD and for CVD as a whole. Their results are based on very much larger numbers of stroke deaths and are more inherently plausible in that stroke and CHD share the same major risk factors. We are not aware of any specific local factors that might explain the difference and so cannot offer any substantive explanation. However, the fact that the effects are consistent across measures suggests that the discrepancy is more likely to be due to the sample than the measures themselves.

One major implication of our results is that the association between intelligence and the major causes of mortality is unlikely to be due to any social, cultural or educational biases that could be ascribed to measures of intelligence.

## 4 Conclusion

Just as intelligence is associated both with all-cause mortality and most of the major causes, so too are reaction times. Effect sizes are of similar magnitude, possibly suggestive of a common cause. The major exception is cancers, particularly those unrelated to smoking, and, in this dataset, stroke.

## Figures and Tables

**Table A1:** Correlation matrix of covariates and cognitive measures

	Sex		SES		Smoking		AH4		CRT mean		SRT mean		CRT SD	
SES	0.09	**												
Smoking	0.18	***	0.20	***										
AH4	−0.05		0.44	***	0.19	***								
CRT mean	0.00		0.24	***	0.15	***	0.50	***						
SRT mean	−0.02		0.15	***	0.05		0.33	***	0.61	***				
CRT SD	−0.18	***	0.10	***	0.10	***	0.28	***	0.49	***	0.21	***		
SRT SD	−0.05		0.11	***	0.05		0.29	***	0.44	***	0.61	***	0.27	***

Coding: Sex 0=female, 1=male; SES 0=non-manual 1=manual; Smoking 1=Never smoked, 2=ex-smoker, 3=current smoker

AH4 scores are reversed

**Table A2:** Results showing effect of adjusting for covariates separately.

		Covariates adjusted for											
		Unadjusted			Sex			Smoking			SES		
Cause of death	Predictor	Hazard ratio	95% CI	p	Hazard ratio	95% CI	p	Hazard ratio	95% CI	p	Hazard ratio	95% CI	p
All Cause	AH4	1.29	1.20 to 1.38	<.001	1.31	1.22 to 1.40	<.001	1.23	1.14 to 1.33	<.001	1.23	1.14 to 1.34	<.001
	CRT mean	1.30	1.22 to 1.38	<.001	1.29	1.21 to 1.38	<.001	1.26	1.17 to 1.35	<.001	1.27	1.18 to 1.35	<.001
	SRT mean	1.23	1.15 to 1.31	<.001	1.23	1.15 to 1.31	<.001	1.22	1.15 to 1.31	<.001	1.21	1.13 to 1.29	<.001
	CRT SD	1.12	1.05 to 1.20	0.001	1.17	1.10 to 1.25	<.001	1.08	1.01 to 1.16	0.025	1.10	1.03 to 1.18	0.006
	SRT SD	1.18	1.11 to 1.26	<.001	1.20	1.12 to 1.28	<.001	1.18	1.10 to 1.26	<.001	1.17	1.09 to 1.25	<.001
CVD	AH4	1.30	1.15 to 1.48	<.001	1.34	1.18 to 1.52	<.001	1.25	1.10 to 1.42	<.001	1.25	1.09 to 1.43	0.001
	CRT mean	1.29	1.15 to 1.44	<.001	1.28	1.14 to 1.43	<.001	1.24	1.10 to 1.40	<.001	1.25	1.11 to 1.41	<.001
	SRT mean	1.21	1.08 to 1.35	<.001	1.21	1.08 to 1.35	<.001	1.20	1.07 to 1.35	0.002	1.18	1.06 to 1.33	0.004
	CRT SD	1.13	1.01 to 1.27	0.039	1.23	1.10 to 1.37	<.001	1.09	0.97 to 1.22	0.159	1.11	0.99 to 1.25	0.078
	SRT SD	1.16	1.03 to 1.30	0.011	1.19	1.06 to 1.33	0.003	1.15	1.03 to 1.29	0.017	1.14	1.02 to 1.28	0.022
CHD	AH4	1.35	1.15 to 1.59	<.001	1.39	1.19 to 1.63	<.001	1.29	1.09 to 1.52	0.003	1.27	1.06 to 1.51	0.008
	CRT mean	1.35	1.18 to 1.55	<.001	1.35	1.17 to 1.54	<.001	1.31	1.13 to 1.52	<.001	1.31	1.13 to 1.52	<.001
	SRT mean	1.25	1.09 to 1.43	0.002	1.25	1.09 to 1.43	0.001	1.24	1.08 to 1.43	0.003	1.22	1.06 to 1.40	0.007
	CRT SD	1.19	1.03 to 1.38	0.020	1.30	1.13 to 1.49	<.001	1.14	0.99 to 1.32	0.075	1.16	1.01 to 1.35	0.043
	SRT SD	1.21	1.05 to 1.39	0.008	1.24	1.08 to 1.43	0.002	1.20	1.04 to 1.38	0.012	1.19	1.03 to 1.37	0.016
Stroke	AH4	1.08	0.84 to 1.38	0.551	1.10	0.86 to 1.41	0.442	1.03	0.79 to 1.32	0.848	1.01	0.77 to 1.32	0.943
	CRT mean	1.10	0.85 to 1.43	0.469	1.09	0.84 to 1.42	0.525	1.05	0.79 to 1.39	0.738	1.06	0.81 to 1.40	0.663
	SRT mean	1.08	0.83 to 1.40	0.589	1.08	0.83 to 1.40	0.567	1.07	0.82 to 1.40	0.629	1.05	0.80 to 1.38	0.702
	CRT SD	0.95	0.73 to 1.24	0.716	1.02	0.79 to 1.32	0.885	0.92	0.71 to 1.20	0.557	0.94	0.72 to 1.22	0.626
	SRT SD	1.02	0.79 to 1.32	0.875	1.04	0.80 to 1.35	0.770	1.02	0.78 to 1.32	0.903	1.01	0.78 to 1.31	0.942
Cancer: all	AH4	1.20	1.07 to 1.36	0.002	1.21	1.07 to 1.37	0.002	1.15	1.02 to 1.30	0.026	1.16	1.02 to 1.33	0.028
	CRT mean	1.13	0.99 to 1.27	0.061	1.12	0.99 to 1.27	0.065	1.07	0.94 to 1.22	0.290	1.09	0.96 to 1.24	0.191
	SRT mean	1.18	1.05 to 1.32	0.005	1.18	1.05 to 1.32	0.005	1.17	1.04 to 1.31	0.009	1.16	1.03 to 1.30	0.014
	CRT SD	1.02	0.90 to 1.15	0.751	1.04	0.92 to 1.17	0.547	0.99	0.87 to 1.12	0.838	1.00	0.89 to 1.14	0.943
	SRT SD	1.13	1.01 to 1.27	0.034	1.14	1.01 to 1.27	0.028	1.12	1.00 to 1.26	0.047	1.12	1.00 to 1.25	0.055
Cancer: smoking	AH4	1.28	1.10 to 1.49	0.001	1.29	1.11 to 1.50	<.001	1.18	1.01 to 1.38	0.037	1.18	1.00 to 1.39	0.048
	CRT mean	1.18	1.02 to 1.37	0.025	1.18	1.02 to 1.37	0.029	1.10	0.93 to 1.29	0.261	1.12	0.96 to 1.31	0.150
	SRT mean	1.20	1.04 to 1.37	0.010	1.20	1.05 to 1.37	0.009	1.18	1.03 to 1.37	0.020	1.16	1.01 to 1.34	0.036
	CRT SD	1.09	0.94 to 1.26	0.270	1.13	0.97 to 1.30	0.109	1.03	0.89 to 1.19	0.692	1.06	0.91 to 1.23	0.444
	SRT SD	1.20	1.05 to 1.37	0.008	1.21	1.06 to 1.38	0.005	1.19	1.04 to 1.37	0.013	1.18	1.03 to 1.35	0.018
Cancer: other	AH4	1.08	0.88 to 1.32	0.453	1.07	0.88 to 1.31	0.483	1.10	0.90 to 1.35	0.351	1.12	0.90 to 1.40	0.305
	CRT mean	1.02	0.81 to 1.27	0.891	1.02	0.82 to 1.27	0.874	1.03	0.83 to 1.29	0.784	1.03	0.82 to 1.29	0.810
	SRT mean	1.13	0.93 to 1.38	0.229	1.13	0.92 to 1.38	0.232	1.14	0.93 to 1.39	0.210	1.14	0.93 to 1.39	0.203
	CRT SD	0.90	0.72 to 1.12	0.331	0.88	0.70 to 1.10	0.250	0.90	0.72 to 1.13	0.365	0.90	0.72 to 1.12	0.348
	SRT SD	0.99	0.80 to 1.23	0.962	0.99	0.80 to 1.23	0.930	1.00	0.81 to 1.24	0.993	1.00	0.80 to 1.24	0.988
Respiratory	AH4	1.52	1.23 to 1.88	<.001	1.54	1.24 to 1.91	<.001	1.42	1.14 to 1.78	0.002	1.41	1.12 to 1.79	0.004
	CRT mean	1.49	1.27 to 1.75	<.001	1.48	1.26 to 1.74	<.001	1.46	1.21 to 1.75	<.001	1.45	1.22 to 1.72	<.001
	SRT mean	1.33	1.12 to 1.58	0.001	1.33	1.12 to 1.57	0.001	1.33	1.11 to 1.58	0.002	1.29	1.08 to 1.55	0.004
	CRT SD	1.10	0.90 to 1.35	0.336	1.15	0.94 to 1.41	0.171	1.04	0.85 to 1.28	0.679	1.07	0.87 to 1.31	0.508
	SRT SD	1.38	1.18 to 1.63	<.001	1.40	1.19 to 1.65	<.001	1.39	1.18 to 1.65	<.001	1.37	1.16 to 1.61	<.001
Digestive	AH4	1.33	0.97 to 1.83	0.079	1.34	0.98 to 1.85	0.070	1.29	0.93 to 1.80	0.129	1.33	0.93 to 1.90	0.113
	CRT mean	1.32	0.99 to 1.75	0.055	1.31	0.99 to 1.74	0.058	1.29	0.96 to 1.75	0.095	1.30	0.97 to 1.75	0.075
	SRT mean	1.20	0.90 to 1.61	0.212	1.20	0.90 to 1.61	0.209	1.20	0.89 to 1.62	0.231	1.19	0.88 to 1.60	0.252
	CRT SD	1.41	1.09 to 1.83	0.010	1.45	1.12 to 1.87	0.005	1.37	1.06 to 1.79	0.018	1.40	1.08 to 1.82	0.012
	SRT SD	1.33	1.03 to 1.71	0.031	1.34	1.03 to 1.73	0.026	1.33	1.02 to 1.73	0.033	1.32	1.02 to 1.71	0.036
Dementia	AH4	1.24	0.89 to 1.72	0.198	1.24	0.89 to 1.72	0.208	1.21	0.86 to 1.69	0.269	1.09	0.76 to 1.57	0.638
	CRT mean	1.28	0.94 to 1.74	0.119	1.28	0.94 to 1.75	0.114	1.25	0.91 to 1.72	0.161	1.21	0.86 to 1.69	0.272
	SRT mean	1.17	0.83 to 1.63	0.369	1.17	0.83 to 1.63	0.371	1.16	0.83 to 1.63	0.391	1.12	0.79 to 1.59	0.511
	CRT SD	1.07	0.77 to 1.49	0.675	1.06	0.76 to 1.48	0.736	1.06	0.76 to 1.46	0.745	1.03	0.74 to 1.44	0.845
	SRT SD	1.02	0.72 to 1.45	0.900	1.02	0.72 to 1.44	0.919	1.02	0.72 to 1.44	0.921	1.00	0.70 to 1.43	0.989
Other Causes	AH4	1.30	1.04 to 1.63	0.020	1.32	1.06 to 1.65	0.015	1.27	1.00 to 1.60	0.046	1.28	1.00 to 1.64	0.049
	CRT mean	1.62	1.38 to 1.89	<.001	1.60	1.37 to 1.87	<.001	1.63	1.38 to 1.92	<.001	1.61	1.37 to 1.89	<.001
	SRT mean	1.39	1.17 to 1.65	<.001	1.39	1.17 to 1.64	<.001	1.39	1.17 to 1.67	<.001	1.38	1.15 to 1.64	<.001
	CRT SD	1.32	1.09 to 1.60	0.005	1.37	1.13 to 1.65	0.001	1.28	1.06 to 1.56	0.011	1.30	1.07 to 1.58	0.007
	SRT SD	1.18	0.96 to 1.44	0.115	1.19	0.97 to 1.46	0.091	1.18	0.96 to 1.44	0.124	1.17	0.95 to 1.43	0.138

AH4 scores are reversed.

**Table A3:** Results for cancers broken down into lung cancer and other cancers

		Unadjusted			Sex, smoking, SES adjusted			Fully adjusted			Selected		
Cause of Death	Predictor	Hazard ratio	95% CI	p	Hazard ratio	95% CI	p	Hazard ratio	95% CI	p	Hazard ratio	95% CI	p
Lung Cancer	AH4	1.38	1.10 to 1.74	0.005	1.13	0.86 to 1.47	0.376	1.15	0.85 to 1.54	0.362			
	CRT mean	1.13	0.90 to 1.42	0.294	0.93	0.71 to 1.22	0.593	0.75	0.52 to 1.09	0.136			
	SRT mean	1.19	0.97 to 1.46	0.096	1.14	0.91 to 1.42	0.247	1.05	0.76 to 1.44	0.771			
	CRT SD	1.00	0.80 to 1.26	0.996	0.92	0.73 to 1.16	0.484	0.93	0.72 to 1.21	0.600			
	SRT SD	1.30	1.08 to 1.57	0.005	1.28	1.05 to 1.56	0.013	1.36	1.06 to 1.74	0.017	1.28	1.05 to 1.56	0.013
Other Cancers	AH4	1.15	1.01 to 1.33	0.042	1.15	0.98 to 1.34	0.089	1.12	0.94 to 1.33	0.215			
	CRT mean	1.13	0.98 to 1.31	0.090	1.11	0.95 to 1.29	0.174	0.99	0.79 to 1.24	0.914			
	SRT mean	1.18	1.03 to 1.34	0.017	1.17	1.02 to 1.34	0.023	1.22	0.99 to 1.49	0.064	1.17	1.02 to 1.34	0.023
	CRT SD	1.01	0.87 to 1.16	0.905	1.00	0.86 to 1.16	0.983	0.97	0.82 to 1.15	0.738			
	SRT SD	1.04	0.91 to 1.20	0.547	1.04	0.90 to 1.20	0.594	0.91	0.75 to 1.10	0.341			

Notes: Unadjusted and covariated adjusted results are from separate models for each cognitive variable. The fully adjusted results are for a single model (per cause) that includes the covariates and all cognitive variables. The Selected model results are derived from the fully adjusted model by backwards elimination of non-significant cognitive variables whilst retaining the covariates.

AH4 scores are reversed.

**Table A4:** Comparison of results with previous studies

		NHANES-III	HALS	Twenty07
CVD	CRT mean		1.19	1.30
	CRT SD		1.06	1.12
	SRT mean	1.36	1.10	1.23
	SRT SD	1.50	1.08	1.18
CHD	CRT mean		1.19	1.35
	CRT SD		1.07	1.19
	SRT mean		1.13	1.25
	SRT SD		1.09	1.21
Stroke	CRT mean		1.29	1.10
	CRT SD		1.06	0.95
	SRT mean		1.13	1.08
	SRT SD		1.18	1.02
Respiratory	CRT mean		1.21	1.49
	CRT SD		1.15	1.10
	SRT mean		1.10	1.33
	SRT SD		1.13	1.38
Cancer all	CRT mean			
	CRT SD			
	SRT mean	0.85		1.18
	SRT SD	0.99		1.13
Lung Cancer	CRT mean		1.20	1.13
	CRT SD		1.08	1.00
	SRT mean		1.06	1.19
	SRT SD		1.06	1.30
